# Genomic epidemiology of the SARS-CoV-2 epidemic in Brazil

**DOI:** 10.1038/s41564-022-01191-z

**Published:** 2022-08-18

**Authors:** Marta Giovanetti, Svetoslav Nanev Slavov, Vagner Fonseca, Eduan Wilkinson, Houriiyah Tegally, José Salvatore Leister Patané, Vincent Louis Viala, Emmanuel James San, Evandra Strazza Rodrigues, Elaine Vieira Santos, Flavia Aburjaile, Joilson Xavier, Hegger Fritsch, Talita Emile Ribeiro Adelino, Felicidade Pereira, Arabela Leal, Felipe Campos de Melo Iani, Glauco de Carvalho Pereira, Cynthia Vazquez, Gladys Mercedes Estigarribia Sanabria, Elaine Cristina de Oliveira, Luiz Demarchi, Julio Croda, Rafael dos Santos Bezerra, Loyze Paola Oliveira de Lima, Antonio Jorge Martins, Claudia Renata dos Santos Barros, Elaine Cristina Marqueze, Jardelina de Souza Todao Bernardino, Debora Botequio Moretti, Ricardo Augusto Brassaloti, Raquel de Lello Rocha Campos Cassano, Pilar Drummond Sampaio Corrêa Mariani, João Paulo Kitajima, Bibiana Santos, Rodrigo Proto-Siqueira, Vlademir Vicente Cantarelli, Stephane Tosta, Vanessa Brandão Nardy, Luciana Reboredo de Oliveira da Silva, Marcela Kelly Astete Gómez, Jaqueline Gomes Lima, Adriana Aparecida Ribeiro, Natália Rocha Guimarães, Luiz Takao Watanabe, Luana Barbosa Da Silva, Raquel da Silva Ferreira, Mara Patricia F. da Penha, María José Ortega, Andrea Gómez de la Fuente, Shirley Villalba, Juan Torales, María Liz Gamarra, Carolina Aquino, Gloria Patricia Martínez Figueredo, Wellington Santos Fava, Ana Rita C. Motta-Castro, James Venturini, Sandra Maria do Vale Leone de Oliveira, Crhistinne Cavalheiro Maymone Gonçalves, Maria do Carmo Debur Rossa, Guilherme Nardi Becker, Mayra Presibella Giacomini, Nelson Quallio Marques, Irina Nastassja Riediger, Sonia Raboni, Gabriela Mattoso, Allan D. Cataneo, Camila Zanluca, Claudia N. Duarte dos Santos, Patricia Akemi Assato, Felipe Allan da Silva da Costa, Mirele Daiana Poleti, Jessika Cristina Chagas Lesbon, Elisangela Chicaroni Mattos, Cecilia Artico Banho, Lívia Sacchetto, Marília Mazzi Moraes, Rejane Maria Tommasini Grotto, Jayme A. Souza-Neto, Maurício Lacerda Nogueira, Heidge Fukumasu, Luiz Lehmann Coutinho, Rodrigo Tocantins Calado, Raul Machado Neto, Ana Maria Bispo de Filippis, Rivaldo Venancio da Cunha, Carla Freitas, Cassio Roberto Leonel Peterka, Cássia de Fátima Rangel Fernandes, Wildo Navegantes, Rodrigo Fabiano do Carmo Said, Carlos F. Campelo de A e Melo, Maria Almiron, José Lourenço, Tulio de Oliveira, Edward C. Holmes, Ricardo Haddad, Sandra Coccuzzo Sampaio, Maria Carolina Elias, Simone Kashima, Luiz Carlos Junior de Alcantara, Dimas Tadeu Covas

**Affiliations:** 1 Laboratório de Flavivirus, Fundacao Oswaldo Cruz, Rio de Janeiro, Brazil; 2 Laboratório de Genética Celular e Molecular, Instituto de Ciências Biologicas, Universidade Federal de Minas Gerais, Belo Horizonte, Minas Gerais Brazil; 3 Department of Science and Technology for Humans and the Environment, University of Campus Bio-Medico di Roma, Rome, Italy; 4 Blood Center of Ribeirão Preto, Ribeirão Preto Medical School, University of São Paulo, Ribeirão Preto, São Paulo, Brazil; 5 Butantan Institute, São Paulo, Brazil; 6 Pan American Health Organization (PAHO)/World Health Organization (WHO), Brasilia, Distrito Federal Brazil; 7 Centre for Epidemic Response and Innovation (CERI), School of Data Science and Computational Thinking, Stellenbosch University, Stellenbosch, South Africa; 8 KwaZulu-Natal Research Innovation and Sequencing Platform (KRISP), School of Laboratory Medicine and Medical Sciences, University of KwaZulu–Natal, Durban, South Africa; 9 Laboratório Central de Saúde Pública do Estado de Minas Gerais (LACEN-MG), Fundação Ezequiel Dias, Belo Horizonte, Minas Gerais Brazil; 10 Laboratório Central de Saúde Pública do Estado da Bahia (LACEN-BA), Salvador, Bahia Brazil; 11 Laboratório Central de Salud Pública, Asunción, Paraguay; 12 Instituto Regional de Investigación em Salud, Universidad Nacional del Caaguazú, Caaguazú, Paraguay; 13 Laboratório de Biología Molecular, Hospital Regional de Coronel Oviedo, Ministerio de Salud Pública y Bienestar Social, Asunción, Paraguay; 14 Laboratório Central de Saúde Pública do Estado de Mato Grosso (LACEN-MT), Cuiabá, Mato Grosso Brazil; 15 Laboratório Central de Saúde Pública do Estado de Mato Grosso do Sul (LACEN-MS), Campo Grande, Mato Grosso do Sul Brazil; 16 Universidade Federal do Mato Grosso do Sul, Campo Grande, Mato Grosso do Sul Brazil; 17 Centro de Genômica Funcional da ESALQ, University of São Paulo, Piracicaba, São Paulo Brazil; 18 NGS Soluções Genômicas, Piracicaba, São Paulo Brazil; 19 Mendelics Análise Genômica, São Paulo, Brazil; 20 Instituto de Biologia Molecular, Laboratório Antonello, Rio Grande do Sul, Brazil; 21 Universidade Federal de Ciencias da Saúde de Porto Alegre (UFCSPA), Universidade Feevale, Grupo Exame Laboratórios, Rio Grande do Sul, Brazil; 22 Vigilancia em Saúde do Estado de Mato Grosso, Cuiabá, Mato Grosso Brazil; 23 Secretaria de Saude do Estado do Mato Grosso do Sul, Campo Grande, Mato Grosso do Sul Brazil; 24 Laboratório Central de Saúde Pública do Estado do Paraná (Lacen-PR), Curitiba, Paraná Brazil; 25 Hospital de Clínicas da Universidade Federal do Paraná, Curitiba, Paraná Brazil; 26 Laboratório de Virologia Molecular, Instituto Carlos Chagas/Fiocruz-PR, Curitiba, Paraná Brazil; 27 Department of Bioprocesses and Biotechnology, School of Agricultural Sciences, São Paulo State University (UNESP), Botucatu, São Paulo Brazil; 28 Department of Veterinary Medicine, School of Animal Science and Food Engineering, University of São Paulo, Pirassununga, São Paulo, Brazil; 29 Medicine School of São José do Rio Preto (FAMERP), São José do Rio Preto, São Paulo Brazil; 30 Molecular Biology Laboratory, Applied Biotechnology Laboratory, Clinical Hospital of the Botucatu Medical School, São Paulo, Brazil; 31 Fundação Oswaldo Cruz, Bio-Manguinhos, Rio de Janeiro, Rio de Janeiro, Brazil; 32 Coordenação Geral dos Laboratórios de Saúde Pública/Secretaria de Vigilância em Saúde, Ministério da Saúde, (CGLAB/SVS-MS), Brasília, Distrito Federal Brazil; 33 Coordenação Geral das Arboviroses, Secretaria de Vigilância em Saúde/Ministério da Saúde (CGARB/SVS-MS), Brasília, Distrito Federal Brazil; 34 Departamento de Imunização e Doenças Transmissíveisa/Secretaria de Vigilancia em Saude, Ministerio da Saude, Brasılia, Distrito Federal Brazil; 35 Department of Zoology, University of Oxford, Oxford, UK; 36 Biosystems and Integrative Sciences Institute, Universidade de Lisboa, Lisboa, Portugal; 37 Centre for the AIDS Programme of Research in South Africa (CAPRISA), Durban, South Africa; 38 Department of Global Health, University of Washington, Seattle, WA USA; 39 Marie Bashir Institute for Infectious Diseases and Biosecurity, School of Life and Environmental Sciences and School of Medical Sciences, University of Sydney, Sydney, New South Wales Australia

**Keywords:** SARS-CoV-2, Phylogenetics

## Abstract

The high numbers of COVID-19 cases and deaths in Brazil have made Latin America an epicentre of the pandemic. SARS-CoV-2 established sustained transmission in Brazil early in the pandemic, but important gaps remain in our understanding of virus transmission dynamics at a national scale. We use 17,135 near-complete genomes sampled from 27 Brazilian states and bordering country Paraguay. From March to November 2020, we detected co-circulation of multiple viral lineages that were linked to multiple importations (predominantly from Europe). After November 2020, we detected large, local transmission clusters within the country. In the absence of effective restriction measures, the epidemic progressed, and in January 2021 there was emergence and onward spread, both within and abroad, of variants of concern and variants under monitoring, including Gamma (P.1) and Zeta (P.2). We also characterized a genomic overview of the epidemic in Paraguay and detected evidence of importation of SARS-CoV-2 ancestor lineages and variants of concern from Brazil. Our findings show that genomic surveillance in Brazil enabled assessment of the real-time spread of emerging SARS-CoV-2 variants.

## Abstract (web summary)

The COVID-19 pandemic in Brazil is assessed using phylogenetic and phylogeographic analysis.

## Main

At the end of 2019, a respiratory pathogen designated the Severe Acute Respiratory Syndrome Coronavirus 2 (SARS-CoV-2) emerged in the city of Wuhan, China. Since its identification, the virus has spread rapidly around the world, causing the Coronavirus disease 2019 (COVID-19) pandemic (declared on 11 March 2020), with infected patients overwhelming many healthcare systems^1^. By mid-February 2022, more than 415 million cases of COVID-19 with more than 5.84 million associated deaths, had been reported globally^2^. Brazil is the epicentre of the COVID-19 epidemic in the Americas, with more than 21.2 million cases and a death toll of 591,000 reported cases (September 2021). Brazil is one of the countries hit hardest by COVID-19^3^. Crucially, a lack of genome sequence data from Brazil has limited our ability to fully understand transmission dynamics at the national scale.

We present a phylogenetic and phylogeographic analysis of genomic data from 27 Brazilian states and one neighbouring country, Paraguay, collected up to September 2021. We evaluate the genomic epidemiology of SARS-CoV-2 in Brazil, including the emergence and spread of key viral variants of concern (VOCs; for example, Gamma) and variants under monitoring (VUMs; for example, Zeta), to assess how their emergence may have contributed to a more severe second wave in the country.

## Results

### COVID-19 transmission dynamics in Brazil

**Fig. 1:**
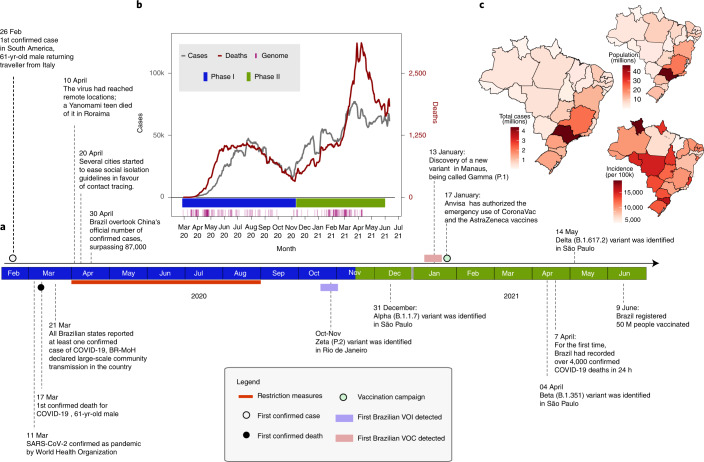
Key events after the first confirmed SARS-CoV-2 infection in Brazil. **a**, Timeline of SARS-CoV-2 key events in Brazil. **b**, Epidemic curve showing the progression of reported daily viral infection numbers in Brazil from the beginning of the epidemic (grey) and deaths (red) in the same period, with restriction phases indicated by the horizontal bar at the bottom. **c**, Map of cumulative SARS-CoV-2 cases per 100,000 inhabitants in Brazil up to June 2021.

The first confirmed infection of SARS-CoV-2 in Brazil was on 26 February 2020 in the State of São Paulo (SP), in a traveller returning from Italy (Fig. 1a). On 17 March 2020, the first COVID-19-related death, a 61-year-old male, was reported in the same state^4,5^. Four days later, all Brazilian states reported at least one confirmed case of COVID-19 and the Brazilian Ministry of Health (BRMoH) declared an outbreak of large-scale community transmission of the virus^6^. By 10 April 2020, the virus had already reached remote locations, such as the Yanomami indigenous community located in the state of Roraima in northern Brazil^6^ (Fig. 1a).

After the World Health Organization (WHO) declared the outbreak of SARS-CoV-2 as a public health emergency of international concern on 30 January 2020, the Brazilian government introduced restriction measures to mitigate viral spread (Fig. 1a)^7^. The primary measure involved social isolation, followed by the closure of schools, universities and non-essential businesses^8^. Additional measures included the mandatory use of personal protective masks^9^, the cancellation of events expected to attract large numbers of people and tourists, and opening only of services considered as essential such as markets and pharmacies^8,10^. However, while the epidemic was growing, restriction measures were progressively eased to mitigate negative impacts on the economy. Notably, even during periods of restriction, travel between Brazilian states largely remained possible, enabling SARS-CoV-2 transmission throughout the country^11^. Travel was probably linked to the emergence of more contagious viral lineages, such as VOC Gamma (lineage P.1) and VUM Zeta (lineage P.2). Notably, these variants may have contributed to a second wave that was more severe in terms of infections and deaths than the first wave (Fig. 1b)^11,12–14^.

The COVID-19 death toll in Brazil rose steadily after March 2021. It reached a daily total of 4,250 deaths on April 2021, the highest number of daily fatalities from COVID-19 worldwide (Fig. 1b). Signs of collapse of the health system were reported in numerous cities around the country. The situation worsened after multiple VOCs and VUMs emerged during a slow vaccination campaign^15^. Vaccination in Brazil began on 17 January 2021, when the Instituto Butantan imported the first 6 million doses of CoronaVac (a whole-virus inactivated vaccine) from Sinovac Biotech (Fig. 1a)^16,17^. As of 16 February 2022, approximately 71.8% of the Brazilian population had been vaccinated with the first dose of any of the vaccines available (CoronaVac, AstraZeneca, Pfizer and Janssen), but only 22% were fully vaccinated (with a single dose of Janssen or two doses of any other vaccine)^18^.

By analysing the total number of COVID-19 notified cases to the end of September 2021, we observed that the Brazilian region with the highest population density (Southeast) also contained the highest number of the cases registered in the country, with the state of São Paulo documenting the largest number of cases (*n* = 4,369,410) in that period (Fig. 1c). However, when we considered the incidence rate (number of reported cases per population) by state, we found that the Midwest, the least populated region in Brazil, had the highest incidence rate, with 13,604.23 cases per 100,000 inhabitants^1^.

### SARS-CoV-2 genomic data

A total of 3,866 near-full genome sequences from SARS-CoV-2 RT–qPCR positive samples were obtained as part of this study. SARS-CoV-2 sequencing spanned February 2020 to June 2021, with samples from 8 of the 27 Brazilian states (São Paulo, 3,309; Rio Grande do Sul, 48; Paraná, 55; Minas Gerais, 80; Mato Grosso do Sul, 36; Mato Grosso, 51; Bahia, 224) and one neighbouring country, Paraguay (*n* = 63). Almost half of the sequences were from Southeast Brazil, comprising the states of São Paulo and Rio de Janeiro that reported the most cases (Fig. 1c)^6^. Sequenced genomes were from samples collected from 2,023 females and 1,843 males (Supplementary Tables 1 and 2), with a median age of 41.72 years (range: 1 to 90 years of age). All tested samples contained sufficient viral genetic material (≥2 ng µl^−1^) for library preparation. For positive samples, PCR cycle threshold (Ct) values were on average 19.93 (range: 10.75–30). Sequences had a median genome coverage of 95% (range: 80–99.99) and average genome coverage was typically higher for samples with lower Ct values (Supplementary Fig. 1). Epidemiological information and sequencing statistics of the generated sequences from Brazil and Paraguay are reported in Supplementary Tables 1 and 2, respectively. Sequences were assigned to 39 different PANGO lineages on the basis of the proposed dynamic nomenclature for SARS-CoV-2 lineages (Supplementary Fig. 1, and Tables 1 and 2) and have been submitted to GISAID following the WHO guidelines (Supplementary Tables 1 and 2) (Pangolin version 3.1.7, August 2021).

### Phylogenetic inference and lineage diversity

**Fig. 2:**
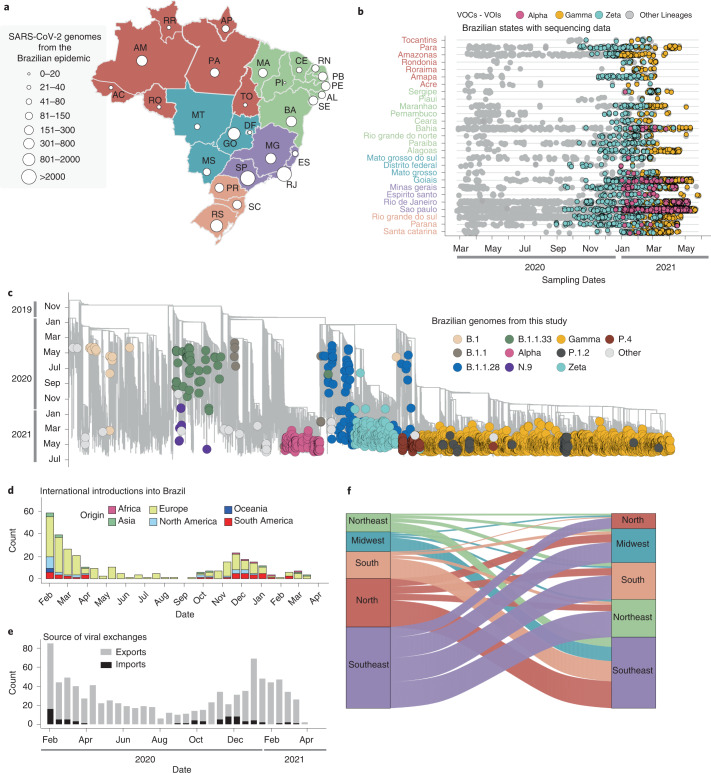
Phylogenetic analysis and SARS-CoV-2 lineage dynamics in Brazil. **a**, Map of Brazil with the number of sequences in GISAID as of 30 June 2021. The map is coloured according to geographical macro region: North (red), Northeast (green), Southeast (purple), Midwest (light blue) and South (light orange). AC, Acre; AL, Alagoas; AP, Amapá; AM, Amazonas; BA, Bahia; CE, Ceará; DF, Distrito Federal; ES, Espírito Santo; GO, Goiás; MA, Maranhão; MT, Mato Grosso; MS, Mato Grosso do Sul; MG, Minas Gerais; PA, Pará; PB, Paraíba; PR, Paraná; PE, Pernambuco; PI, Piauí; RR, Roraima; RO, Rondônia; RJ, Rio de Janeiro; RN, Rio Grande do Norte; RS, Rio Grande do Sul; SC, Santa Catarina; SP, São Paulo; SE Sergipe; TO, Tocantins. **b**, Temporal sampling of sequences in Brazilian states through time with VOCs highlighted and annotated according to their PANGO lineage assignment. **c**, Time-resolved maximum-likelihood phylogeny containing high-quality near-full genome sequences from Brazil (*n* = 3,866) obtained from this study, analysed against a backdrop of global reference sequences (*n* = 25,288). VUMs and VOCs are highlighted on the phylogeny. **d**, Sources of viral introductions into Brazil characterized as external introductions from the rest of the world. **e**, Sources of viral exchanges (imports and exports) into and out of Brazil. **f**, Number of viral exchanges within Brazilian regions by counting the state changes from the root to the tips of the phylogeny in **c**.

The rapid spread of SARS-CoV-2, together with the reported circulation of several VOCs and VUMs in Brazil, prompted an intensification of genomic surveillance by the National Network for Pandemic Alert of SARS-CoV-2 at the end of December 2020. As of 30 June 2021, more than 17,135 SARS-CoV-2 genomes from all 27 Brazilian states had been deposited in the GISAID database (Fig. 2a). The states with the highest number of sequenced genomes were São Paulo (*n* = 9,600) and Rio de Janeiro (*n* = 2,031). Although genomic surveillance began as soon as the first confirmed infections were detected in Brazil, by the end of June 2021 there was still a paucity of genomic data from some states, such as Roraima (*n* = 29), Acre (*n* = 29), Rondônia (*n* = 37), Tocantins (*n* = 27), Piauí (*n* = 19) and the Federal District (*n* = 33) (Fig. 2a). Half of all Brazilian genomes were deposited in early 2021, suggesting that surveillance was at its peak in the second wave following the emergence of Gamma (and other VOCs (for example, Alpha/B.1.1.7)) and VUMs (for example, Zeta) throughout the country (Fig. 2b).

**Fig. 3:**
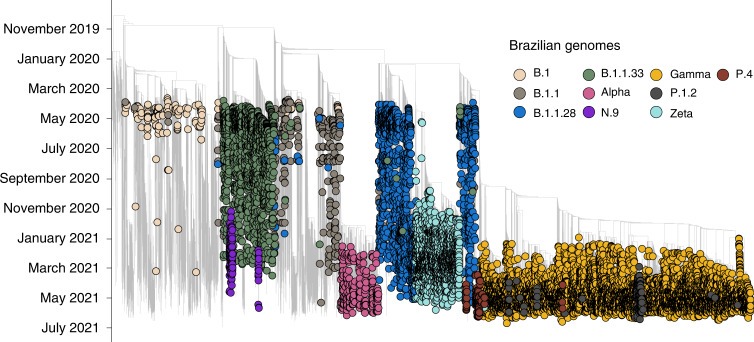
Fully annotated Brazilian SARS-CoV-2 time tree. Time-resolved maximum-likelihood phylogeny containing 17,135 high-quality Brazilian SARS-CoV-2 near-full-genome sequences (*n* = 3,866 generated in this study) analysed against a backdrop of global reference sequences. VUMs and VOCs are highlighted.

To understand the dynamics of SARS-CoV-2 spread in Brazil, we coupled epidemiological data with phylodynamic analysis for a data set comprising 25,288 available globally representative genomes, including the genomes sequenced in this study (*n* = 3,866) sampled from 26 December 2019 to 28 June 2021 (Figs. 2c and 3). A date-stamped phylogeny of these data indicated that most of the Brazilian sequences were interspersed with those introduced from several countries (Figs. 2c,d). This pattern further indicated that the co-circulation of multiple SARS-CoV-2 lineages over time was linked to multiple importations followed by large local transmissions concomitant with a high number of infections (Fig. 2c,d).

Using an ancestral location state reconstruction on the dated phylogeny, we were able to infer the number of viral imports and exports between Brazil and the rest of the world, and between individual Brazilian regions (hereafter referred as the North, Northeast, Midwest, Southeast and South regions) (Fig. 2d–f). The bulk of imported introductions (estimated to be 114 independent ones) were largely from Europe (Fig. 2d), occurring before the implementation of restriction measures (April 2020) when the epidemic was rapidly progressing (Fig. 2d,e). However, at least 33 introduction events were inferred to have occurred during enforcement of preventive measures up to August 2020 (Fig. 2d,e), and hence before those measures were loosened. Finally, although Brazil was a major virus importer, there were approximately 10 times more inferred exportation events out of Brazil than viral introductions into Brazil (Fig. 2e).

Our estimates of viral movement within Brazil further suggested that the Southeast region was the largest contributor of viral exchanges to other regions, comprising approximately 40% of viral movements from one geographical region to another, followed by the North region that contributed to approximately 25% of all viral movements. Although these estimates are in line with epidemiological data, this observation is probably also influenced by these two regions having the greatest number of sequences available for analysis.

### Spatiotemporal spread of Gamma and Zeta variants in Brazil

We next focused on two variants (Gamma/P.1 and Zeta/P.2) that evolved from the B.1.1.28 lineage and grew into large transmission clusters during the second wave of the epidemic in Brazil after January 2021. To assess the detailed evolution of these lineages over time, we performed a spatiotemporal phylogeographic analysis using a molecular clock model.

**Fig. 4:**
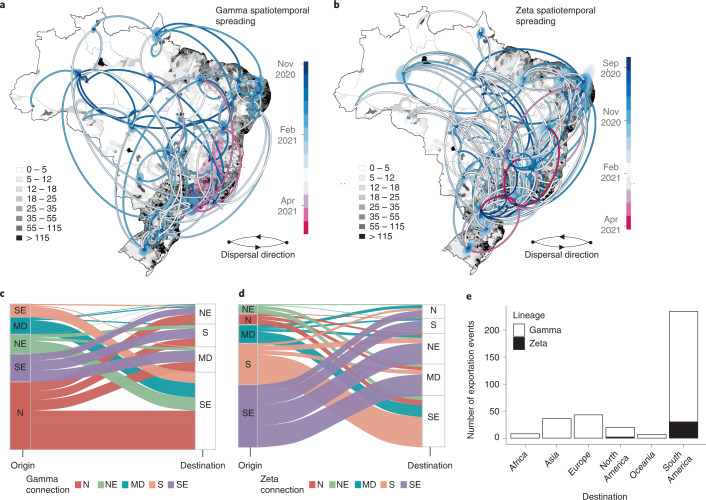
Spatiotemporal spread of VOCs and VUMs in Brazil. **a**, Phylogeographic reconstruction of the spread of the Gamma VOC in Brazil. Circles represent nodes of the maximum clade credibility phylogeny and are coloured according to their inferred time of occurrence. Shaded areas represent the 80% highest posterior density interval and depict the uncertainty of the phylogeographic estimates for each node. Solid curved lines denote the links between nodes and the directionality of movement. Differences in population density are shown on a dark-white scale. **b**, Phylogeographic reconstruction of the spread of the Zeta VUM across Brazil. Circles represent nodes of the maximum clade credibility phylogeny and are coloured according to their inferred time of occurrence. Shaded areas represent the 80% highest posterior density interval and depict the uncertainty of the phylogeographic estimates for each node. Solid curved lines denote the links between nodes and the directionality of movement. Differences in population density are shown on a dark-white scale. **c**, Number of exchanges of the Gamma variant between Brazilian regions (N, North; NE, Northeast; MD, Midwest; SE, Southeast; S, South). **d**, Number of exchanges of the Zeta variant between Brazilian regions. **e**, Sources of viral export of the VOC and VUM from Brazil to the rest of the world.

The Gamma VOC was first sampled in Brazil in early January 2021^12,19^. It displayed an unusual number of lineage-defining mutations in the S protein, including three designated that may impact transmission, immune escape and virulence—N501Y, E484K and K417T^20–22^. In line with previous estimates^12,19^, our phylogeographic analysis suggested that the Gamma variant emerged around 21 November 2020 (95% highest posterior density, 12–29 November 2020) in Manaus (Amazonas state) in Northern Brazil and spread extensively among Brazilian regions (Fig. 4a,c). Our data reveal multiple introductions of this lineage from the Amazonas state to Brazil’s southeastern, northeastern and midwestern states (Fig. 4a,c). By mid-January 2021, the southeastern and northern regions had also acted as source populations for the introduction of this variant into the neighbouring southern region (Fig. 4a,c).

Zeta (P.2) is defined by the presence of the S:E484K mutation in the receptor binding domain (RBD) and other lineage-defining mutations outside the S protein^13,14^. Although it was first described in samples from October 2020 in the state of Rio de Janeiro, our phylogeographic reconstruction suggests that the variant originated from Paraná state in South Brazil in late August 2020 (95% highest posterior density, 19 August to 03 September 2020) (Fig. 4b). Since then, Zeta has spread multiple times to much of the southeastern, northeastern, midwestern and northern Brazilian regions (Fig. 4d). Together, our results further suggest that the transmission dynamics roughly followed patterns of population density, moving most often between the most populous localities (Fig. 4a,b).

By estimating the pattern of migration flows, we also examined the potential role of Brazil as an exporter of the Gamma and Zeta variants to the rest of the world (Fig. 4). While the North region seeded approximately 47% of all Gamma infections into other regions, consistent with it being where this lineage originated, there is strong evidence both from phylogeographic analysis (Fig. 4a) and ancestral state reconstruction (Fig. 4c) that there was considerable subsequent transfer of Gamma between all regions. Zeta had a different dispersal pattern from Gamma, with 73% of all Zeta movements originating from the Southeast and South regions, consistent with our phylogeographic reconstruction that this is the geographic source of this lineage (Fig. 4).

Our analysis further revealed that Brazil has contributed to the international spread of both variants, with at least 316 and 32 exportation events to the rest of the world detected for Gamma and Zeta variants, respectively (Fig. 4e). Consistent with importations, most exports were to South America (65%) and Europe (14%), followed by Asia (11%), North America (5%), Africa (2.5%) and Oceania (2.5%), with an increase between January and March 2021 coinciding with the second wave of infections in Brazil and some relaxation of international travel restrictions (Fig. 4e). As shown elsewhere, these results demonstrate that under relaxation of travel restrictions, SARS-CoV-2 lineages can spread to a diverse range of international locations^23–28^.

### Cross-border SARS-CoV-2 transmission from Brazil to Paraguay

**Fig. 5:**
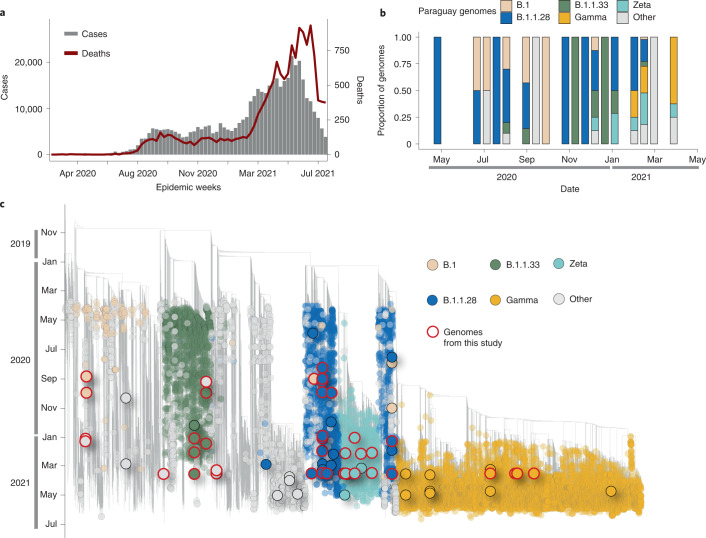
The SARS-CoV-2 epidemic in Paraguay. **a**, Epidemic curve showing the progression of reported viral infection numbers in Paraguay from the beginning of the epidemic (grey) and deaths (red) in the same period. **b**, Progressive distribution of the top 20 PANGO lineages in Paraguay over time. **c**, Time-resolved maximum-likelihood tree containing high-quality near-complete genome sequences from Paraguay (*n* = 63) obtained in this study, analysed against a backdrop of global reference sequences. VUMs and VOCs are highlighted on the phylogeny. Genome sequences from Paraguay obtained in this study are highlighted with red borders.

To explore the burden of the Brazilian SARS-CoV-2 pandemic on other South American countries, we provide a preliminary overview of the SARS-CoV-2 epidemic in Paraguay. The first COVID-19 confirmed case was documented in Paraguay on 7 March 2020 in a 32-year-old man from San Lorenzo, Central Department. Thirteen days later, the first death and the first case of community transmission were also confirmed. COVID-19 cases in Paraguay rose sharply in March (Fig. 5a), resulting in 100% occupancy of intensive care beds, prompting the government to declare a strict quarantine to mitigate the spread of the virus^29,30^. By the end of June 2021, a total number of 460,000 confirmed cases and 15,000 coronavirus-related deaths had been reported in Paraguay^29^.

The COVID-19 epidemic in Paraguay can generally be characterized by three phases: phase I starting from 10 March 2020, characterized by restriction measures; phase II since 4 May 2020, also called ‘intelligent/smart quarantine’ with a gradual return to work and social activities; and phase III implemented since 5 October 2020, known as the ‘COVID way of living’, characterized by the relaxation of the restriction measures and the reopening of national borders and resumption of international flights^30^.

Since the beginning of the epidemic, there has been a paucity of whole-genome sequences from Paraguay, with only *n* = 165 whole-genome sequences available on GISAID by the end of July 2021, about 0.0003% of known cases. This seriously impacts the ability to characterize the molecular epidemiology of SARS-CoV-2 at a regional level. In collaboration with the Pan-American Health Organization and the National Public Health Laboratory of Asunción in Paraguay, we obtained a total of 63 near-complete genome sequences sampled between July 2020 and June 2021, representing ~40% of the currently available genomes from this country. The selection of the samples was based on the Ct value (≤30) and availability of epidemiological metadata, such as date of sample collection, sex, age and municipality of residence. Thus, by applying these inclusion criteria, only 63 positive samples were considered suitable for this study. As expected, we observed the co-circulation of multiple SARS-CoV-2 lineages (Fig. 5b), linked to multiple importations and subsequently characterized by large transmission clusters.

Importantly, our phylogenetic analysis revealed that most of the SARS-CoV-2 variants currently circulating in Paraguay, including lineages B.1.1.28, B.1.1.33, Zeta and Gamma, originally emerged in Brazil (Fig. 5a,b), thus suggesting cross-border transmission from Brazil to Paraguay (Fig. 5b,c). This reinforces the importance of non-pharmaceutical measures in containing and preventing the spread of viral strains into neighbouring countries.

As of 31 July 2021, a total of 78% of available genomic sequences from Paraguay were linked to infections caused by Brazilian variants, with the Gamma VOC being the most prevalent lineage in the country. As genome sequencing is not widespread, it is difficult to determine how widely these variants have spread within Paraguay and to other Latin American countries. However, the abundance of COVID-19 cases in Brazil, a country that shares borders with ten countries, suggests that this risk is probably high.

## Discussion

Genome sequencing and epidemiology have been broadly applied to track the spread and evolution of SARS-CoV-2, enabling informed public health policies to curb infections. Genomic data also reveal features of emerging viruses that patient data alone cannot capture. However, the utility of genomic data is constrained by sparse sampling^31^. Brazil is an important case study, but SARS-CoV-2 sequences from Brazil are only available from a tiny fraction of the number of confirmed cases in the country, which restricts their utility as evidence for public health policies, including control strategies.

To better inform public health policies, we analysed genomic data obtained by sequencing 3,866 SARS-CoV-2 infection cases, confirmed by RT–qPCR, from patients residing in 8 of the 27 Brazilian federal states and the neighbouring country of Paraguay. These were analysed together with *n* = 13,328 and *n* = 102 (up to 20 June 2021) publicly available complete genomes sequenced from both countries, respectively. By combining epidemiological and genomic data, we show how the interplay between the implementation of restriction measures and sustained SARS-CoV-2 transmission have shaped the Brazilian epidemic over 20 months, including the dramatic resurgences in case numbers linked with the emergence of VOCs and VUMs. In particular, we show that multiple independent importations of SARS-CoV-2, predominantly from Europe, occurred in Brazil during the early phase of the epidemic (up to April 2020). We further detected multiple (*n* = 33) international introductions during periods characterized by the enforcement of preventive measures, demonstrating that efforts to prevent importation were not completely successful, as previously reported in other countries^32^.

Our analyses show that during 2020, Brazil transitioned from a viral importer to a viral exporter, with approximately 10 times more exportation than importation events in Brazil being inferred (Fig. 2e). Importantly, Brazil became a virus exporter at the same time that VOCs and VUMs were detected. Although we were unable to measure passenger traffic in and out of Brazil, the viral migration pattern that we observed seems to be consistent with results obtained from other studies that combined viral genetic data with epidemiological and travel data. Taken together, it seems clear that the spread of respiratory viruses is governed by a combination of human mobility and population density^24–28^.

We also provide a preliminary overview of the SARS-CoV-2 epidemic in Paraguay, revealing evidence of spread from Brazil. This spread was probably facilitated by air and road networks that form major transport links between Brazil and other parts of South America. The unhampered spread of VOCs and VUMs into South American countries, such as Paraguay, indicates that the land-border controls to curb the international spread of the virus were largely ineffective and highlights the inherent challenges in screening cross-border travellers to contain the spread of this virus. Our data further suggest that the lifting of national and international travel restrictions at specific times during the Brazilian epidemic was probably responsible for both introduction of SARS-CoV-2 from abroad and within-country transmission, with infected travellers as carriers.

More broadly, our study highlights the utility of SARS-CoV-2 genome sequencing for comprehensive country-wide studies of the epidemiology and spread of emerging viral strains. The current co-circulation of VUMs and VOCs in Brazil, together with the slow vaccine rollout, has important implications for public health in this highly populous and regionally important country^33^. Such epidemiological conditions create the perfect environment for the continued evolution of SARS-CoV-2, potentially enabling the emergence of novel variants of altered phenotype.

## Methods

### Ethics statement

This research was approved by the Ethics Review Committee of the Pan-American Health Organization (PAHOERC.0344.01), the Federal University of Minas Gerais (CEP/CAAE: 32912820.6.1001.5149), the University of São Paulo (CEP/FZEA: 4.780.992) and the Blood Center of Ribeirão Preto (CEP/HCRP-FMRP: 50367721.7.1001.5440), and by the Paraguayan Ministry of Public Health and Social Welfare (MSPyBS/S.G. no. 0944/18). The availability of these samples for research purposes during outbreaks of national concern is allowed under the terms of the 510/2016 Resolution of the National Ethical Committee for Research – Brazilian Ministry of Health (CONEP – Comissão Nacional de Ética em Pesquisa, Ministério da Saúde) that authorizes, without the necessity of informed consent, the use of clinical samples collected in the Brazilian Central Public Health Laboratories to accelerate knowledge building and contribute to surveillance and outbreak response. The samples processed in this study were obtained anonymously from material exceeding that needed for routine diagnosis in Brazilian public health laboratories that belong to the public network within BrMoH.

### Genomic surveillance network

As part of the National Network for Pandemic Alert of SARS-CoV-2, we performed genomic monitoring to rapidly understand the spread of SARS-CoV-2 at the national and cross-border levels. This was performed in collaboration with the Central Laboratory of Public Health (LACEN) from the states of Bahia, Mato Grosso, Mato Grosso do Sul, Minas Gerais, as well as the Blood Center of Ribeirão Preto, the Fiocruz Paraná, the Butantan Institute from the state of São Paulo, the University of São Paulo and the Central Laboratory of Health of Paraguay.

### Sample collection and molecular diagnostic assays

Convenience clinical samples from patients with suspected SARS-CoV-2 infection and residing in 8 of the 27 Brazilian federal states and from Asunción, Paraguay, collected between July 2020 and June 2021 were provided for diagnostic and genome sequencing purposes. Viral RNA was extracted from nasopharyngeal swabs using an automated protocol and tested for SARS-CoV-2 by multiplex real-time PCR assays: (1) the Allplex 2019-nCoV assay (Seegene) targeting the envelope (E), the RNA-dependent RNA polymerase (RdRp) and the nucleocapsid (N) genes; (2) the Charité: SARS-CoV2 (E/RP) assay (Bio-Manguinhos/Fiocruz) targeting the E gene and (3) the GeneFinder COVID-19 Plus RealAmp kit (Osang Healthcare) supplied by the BrMoH, Butantan Institute and the Pan-American Health Organization.

### cDNA synthesis and whole-genome sequencing

Samples were selected for sequencing on the basis of the Ct value (≤30) and availability of epidemiological metadata, such as date of sample collection, sex, age and municipality of residence. The preparation of SARS-CoV-2 genomic libraries was performed using both the Illumina COVIDSeq test following the manufacturer’s instructions and nanopore sequencing using the ARTIC Network primal scheme (https://github.com/artic-network/artic-ncov2019/tree/master/primer_schemes/nCoV-2019/V3)^34^. The normalized libraries were loaded onto a 300-cycle MiSeq Reagent Kit v2 and run on the Illumina MiSeq instrument (Illumina).

Due to regional characteristics and the accessibility of resources of the different Brazilian and Paraguayan laboratories, we also applied field SARS-CoV-2 sequencing using the Oxford Nanopore MinION technology. In this case, the SuperScript IV Reverse Transcriptase kit (Invitrogen) was initially used for complementary DNA (cDNA) synthesis following the manufacturer’s instructions. The cDNA generated was subjected to multiplex PCR sequencing using the Q5 High-Fidelity Hot-Start DNA Polymerase (New England Biolabs) and a set of specific primers designed by the ARTIC Network for sequencing the complete SARS-CoV-2 genome (Artic Network version 3)^34^. PCR conditions have been previously reported^34^. All experiments were performed in a biosafety level-2 cabinet. Amplicons were purified using 1x AMPure XP beads (Beckman Coulter) and quantified on a Qubit 3.0 fluorimeter (ThermoFisher) using Qubit dsDNA HS assay kit (ThermoFisher). DNA library preparation was performed using the ligation dequencing kit LSK109 (Oxford Nanopore Technologies) and the native barcoding kit (NBD104 and NBD114, Oxford Nanopore Technologies). Sequencing libraries were loaded into an R9.4 flow cell (Oxford Nanopore Technologies). In each sequencing run, we used negative controls to prevent and check for possible contamination with less than 2% mean coverage.

### Generation of consensus sequences from Illumina and nanopore

The genome assembly pipeline for Illumina reads involved: (1) read trimming and filtering using Trimmomatic;^35^ (2) minimap2^36^ for read mapping against the reference strain (Wuhan-Hu-1 genome reference – US National Center for Biotechnology Information (NCBI) accession NC_045512.2); (3) samtools^37^ for sorting and indexing; (4) Pilon^38^ for improving the indel detection; (5) bwa mem^39^ for remapping against Pilon’s generated consensus; (6) samtools mpileup to generate alignment quality values; (7) seqtk^40^ to generate a quasi-final genome version; (8) bwa mem for a third round of remapping reads against the quasi-final genome; and (9) samtools depth to assess position depths given the *.bam file from the previous step (nucleotide positions with read depth <5 are denoted as ‘N’).

Oxford Nanopore sequencing raw files were basecalled using Guppy v3.4.5 and barcode demultiplexing was performed using qcat. Consensus sequences were generated by de novo assembling using Genome Detective^41^ that uses DIAMOND to identify and classify candidate viral reads in broad taxonomic units, using the viral subset of the Swissprot UniRef protein database. Candidate reads were next assigned to candidate reference sequences using NCBI blastn and aligned using AGA (Annotated Genome Aligner) and MAFFT. Final contigs and consensus sequences are available as FASTA files.

### Data quality control and global data set collection

To ensure the quality of the genome sequences generated in this study and to guarantee the highest possible phylogenetic accuracy, only genomes >29,000 bp with <1% of ambiguities were considered (*n* = 3,866). Multiple sequence alignment was performed with MAFFT^42^ and a preliminary phylogenetic tree was inferred using the maximum-likelihood method in IQ-TREE2, employing the GTR + I model of nucleotide substitution. Before further phylogenetic analysis, our data set was also assessed both for sequences with low data quality (for example, with assembling issues, sequencing and alignment errors, data annotation errors and sample contamination) and for molecular clock signal (that is, temporal structure) using TempEst v1.5.3^43^.

**Fig. 6:**
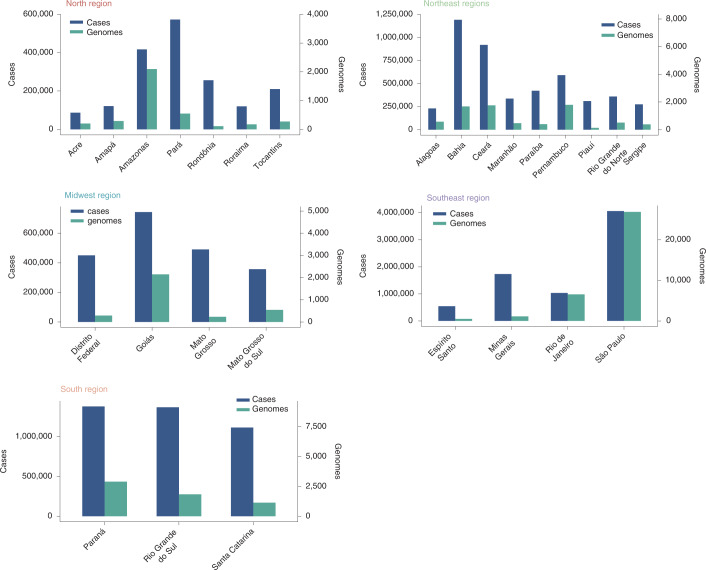
**Number of total cases plotted against the number of sequences available for each Brazilian state/region**.

We appended the 3,866 genome sequences newly generated under this project with an extensive reference data set of SARS-CoV-2 sequences sampled globally and collected since the start of the outbreak, including *n* = 13,328 near-complete genomes from Brazil and *n* = 102 from Paraguay (sampled up to June 30, 2021). A unique set of external references (*n* = 7,992) was obtained by including all the non-Brazilian and non-Paraguayan sequences from the global and South American NextStrain builds (date of access, 30 June 2021). These global references were further supplemented by the inclusion of a random sample of P.1 and P.2 lineages from around the world. This approach was taken to ensure that we captured sufficient global sequence diversity while simultaneously enriching our data set with sequences from South American countries with whom Brazil and Paraguay share borders. Additionally, during our downsampling strategy, we took into account that the different sequencing efforts employed by different Brazilian states resulted in differing numbers of available genomes for each state/region. For this reason, we selected reference sequences considering both the number of reported cases for each state and the number of available genome sequences, aiming at building a more representative data set (Fig. 6).

### Phylogenetic analysis

Sequences were aligned using MAFFT^42^ and submitted to IQ-TREE2 for maximum-likelihood (ML) phylogenetic analysis^44^, employing the general time reversible model of nucleotide substitution and a proportion of invariable sites (+I) as selected by the ModelFinder application. Branch support was assessed using the approximate likelihood-ratio test based on the bootstrap and the Shimodaira–Hasegawa-like procedure with 1,000 replicates.

The raw ML tree topology was then used to estimate the number of viral transmission events between various Brazilian regions and the rest of the world. TreeTime^45^ was used to transform this ML tree topology into a dated tree using a constant mean rate of 8.0 × 10^−4^ nucleotide substitutions per site per year, after the exclusion of outlier sequences. A migration model was fitted to the resulting time-scaled phylogenetic tree in TreeTime, mapping country and regional locations to tips and internal nodes^45^. Using the resulting annotated tree topology, we were able to count the number of transitions (that is, virus importations and exportations) within different Brazilian regions and between Brazil and the rest of the world. Importantly, this analysis was not dependent on a monophyletic clustering of SARS-CoV-2 in Brazil. To provide a measure of confidence in the time and source of viral transitions, we performed the discrete ancestral state reconstruction on 10 bootstrap replicate trees (Supplementary Figs. 2–4).

### Lineage classification

We used the dynamic lineage classification as specified in the Phylogenetic Assignment of Named Global Outbreak LINeages (Pangolin version 3.1.7) protocol^46^. This was aimed at identifying the most epidemiologically important lineages of SARS-CoV-2 circulating within South America (Brazil and Paraguay). Both VOCs and VUMs were designated on the basis of the World Health Organization framework as of July 2021.

### Phylogeographic reconstruction

VOCs and VUMs that emerged in Brazil (that is, P.1 and P.2) were identified as monophyletic groups on the time-resolved phylogenetic trees (Fig. 2 and Supplementary Fig. 5). Genome sequences from these lineages were then extracted to infer continuous phylogeography histories using the Markov chain Monte Carlo (MCMC) method in BEAST v1.10.4^47^, employing the HKY + Γ _nucleotide substitution model and a strict molecular clock in BEAST v.1.10.4 that assumes constant evolutionary rates throughout the phylogeny.

Due to the huge size of the largest clusters, we downsampled them to <600 taxa per clade to infer phylogeographies within a manageable time frame. Accordingly, we retained the ten earliest sequences from each unique sampling location within Brazil and a random distribution over the remaining samples from each location (*n* = 531 for P.1 and *n* = 428 for P.2). Briefly, sequences from the subsampled cluster were aligned using MAFFT and preliminary ML trees were inferred in IQ-TREE2 as described above. Before phylogeographic analysis, each lineage was also assessed for molecular clock signal using the root-to-tip regression method available in TempEst v1.5.3^43^ following the removal of potential outliers that may violate the molecular clock assumption. We accepted temporal structure when the correlation coefficient was >0.2^48^. Linear regression of root-to-tip genetic distances against sampling dates indicated that the SARS-CoV-2 sequences from Gamma and Zeta evolved in a relatively clock-like manner (*r*^2^ = 0.5; correlation coefficient, 0.45 for Gamma and *r*^2^ = 0.5; correlation coefficient, 0.40 for Zeta) (Supplementary Fig. 5).

We modelled the phylogenetic diffusion and spread of these lineages within Brazil by analysing localized transmission (between Brazilian regions) using a flexible relaxed random walk diffusion model^49,50^ that accommodates branch-specific variation in rates of dispersal, with a Cauchy distribution and a jitter window site of 0.01^50,51^. The choice of Cauchy distribution was based on recent studies demonstrating that it is successful for this kind of analyses based on genomes of SARS-CoV-2 and its variants^4,48,52–54^. For each sequence, latitude and longitude coordinates were attributed. MCMC analyses were set up in BEAST v1.10.4, running in duplicate for 100 million interactions and sampling every 10,000 steps in the chain. Convergence for each run was assessed in Tracer v1.7.1 (effective sample size for all relevant model parameters >200). Maximum clade credibility trees for each run were summarized using TreeAnnotator after discarding the initial 10% as burn-in. While the sampling is relatively homogeneous among sampled locations, the phylogeographic reconstruction will of course remain sampling-dependent. Hence, differences in sampling effort may have impacted the estimated transition frequencies between locations. Despite this caveat, the phylogeographic analysis still provides important information on the history and dynamics of dispersal of the lineages sampled, which can in turn provide insights into the connectivity of locations along the transmission network. Finally, we used the R package ‘seraphim’^53^ to extract and map spatiotemporal information embedded in the posterior trees. Note that a transmission link on the phylogeographic map can denote one or more transmission events depending on the phylogeographic inference.

### SARS-CoV-2 time series from Brazil

Data from weekly notified and laboratory confirmed cases of infection by SARS-CoV-2 in Brazil were supplied by the Brazilian Ministry of Health, as made available by the COVIDA network at https://github.com/wcota/covid19br. For convenience, the geographical locations were aggregated by Brazilian macro regions: North, Northeast, Southeast, South and Midwest.

### Reporting summary

Further information on research design is available in the Nature Research Reporting Summary linked to this article.

## Supplementary information

Supplementary Information Supplementary Figs. 1–5.

Reporting Summary

Supplementary Table 1 Information on the n = 3,803 SARS-CoV-2 Brazilian samples sequenced as part of this study.

Supplementary Table 2 Information on the n = 63 SARS-CoV-2 from Paraguay samples sequenced as part of this study.

Supplementary Table 3 GISAID acknowledgment table.

## Data Availability

All sequences that were generated and used in the present study are listed in Supplementary Tables 1–3 (accessible on the GitHub repository) along with their GISAID sequence IDs, dates of sampling, the originating and submitting laboratories and main authors. All input files (for example, alignments or XML files), all resulting output files and scripts used in the study are shared publicly on GitHub (https://github.com/genomicsurveillance/Genomic_epidemiology_reveals_how_restriction_measures_shaped_the_SARS-CoV-2_epidemic_in_Brazil).
