# Supplementary material for: Genomic epidemiology of the SARS-CoV-2 epidemic in Brazil
Source: Nat Microbiol. 2022 Aug 18;7(9):1490–500. doi: 10.1038/s41564-022-01191-z (PMC9417986; doi:10.1038/s41564-022-01191-z)

nature portfolio

https://doi.org/10.1038/s41564-022-01191-z

Supplementary information

Genomic epidemiology of the SARS-CoV-2 epidemic in Brazil

In the format provided by the authors and unedited

Supplementary figure 1.

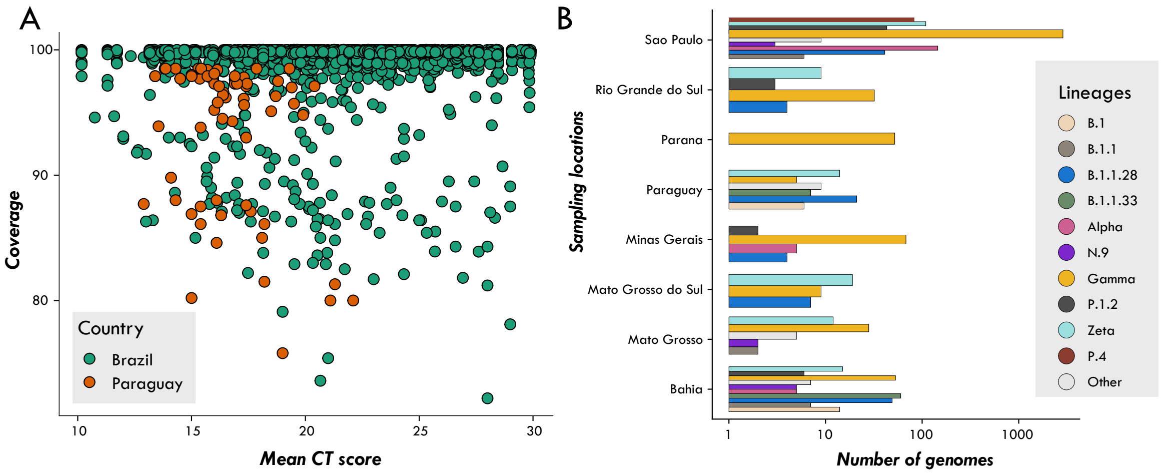

Supplementary figure 2.

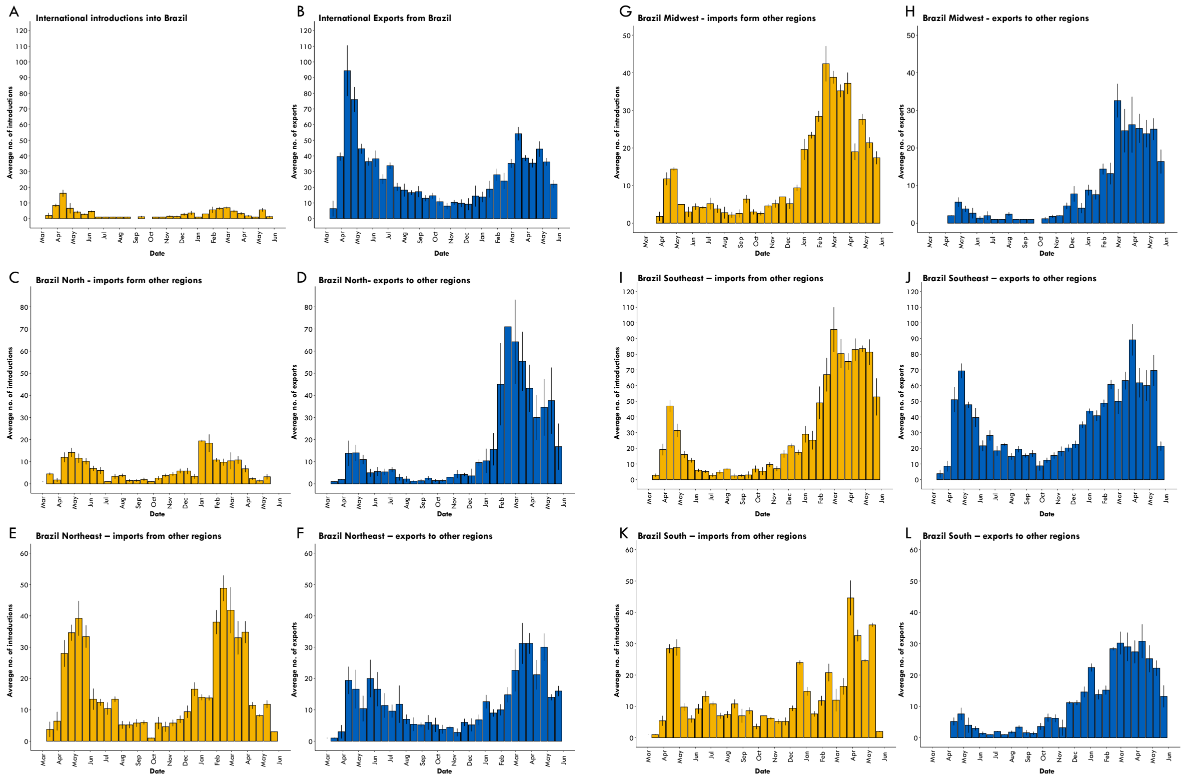

Supplementary figure 3.

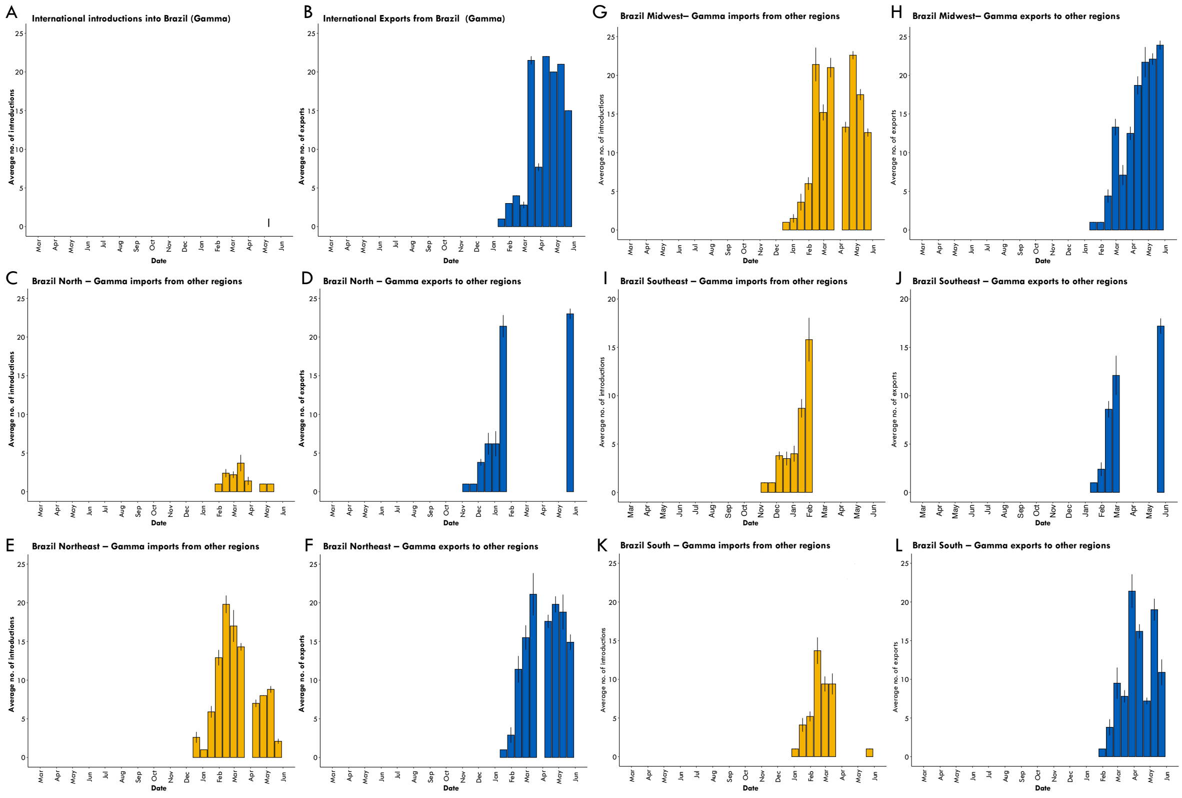

Supplementary figure 4.

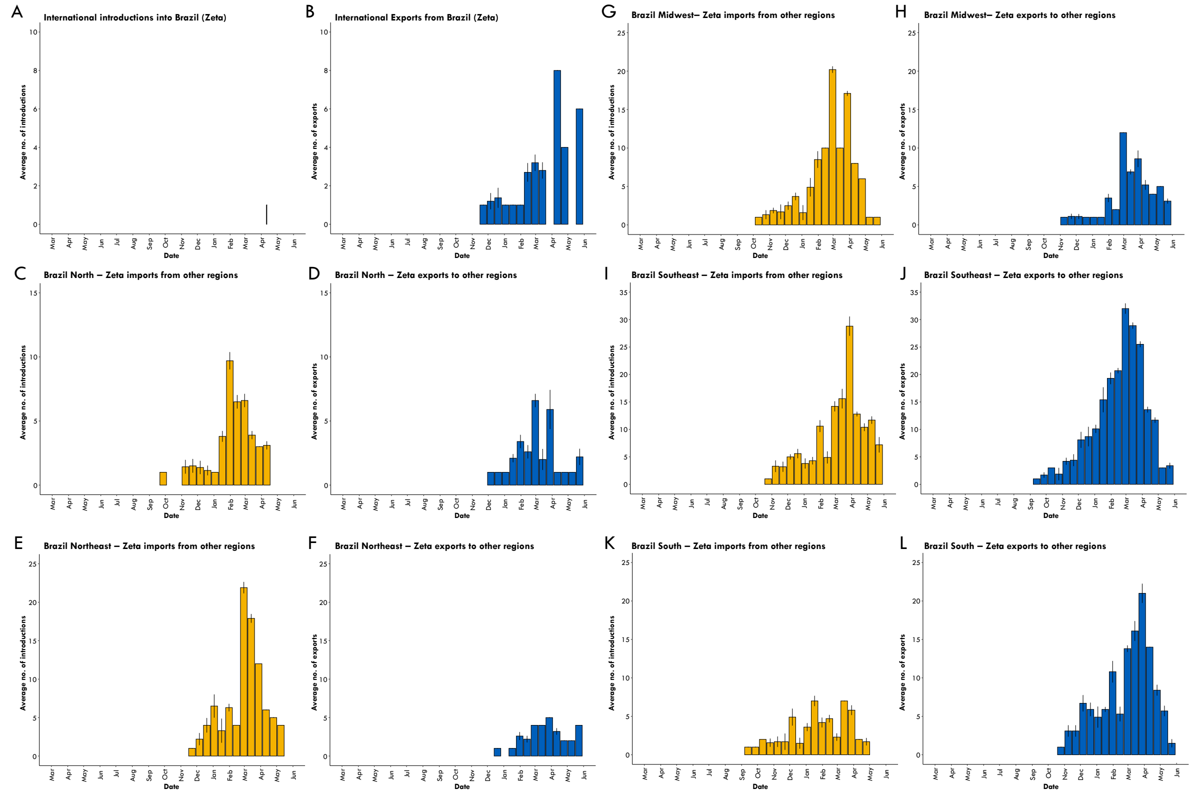

Supplementary figure 5.

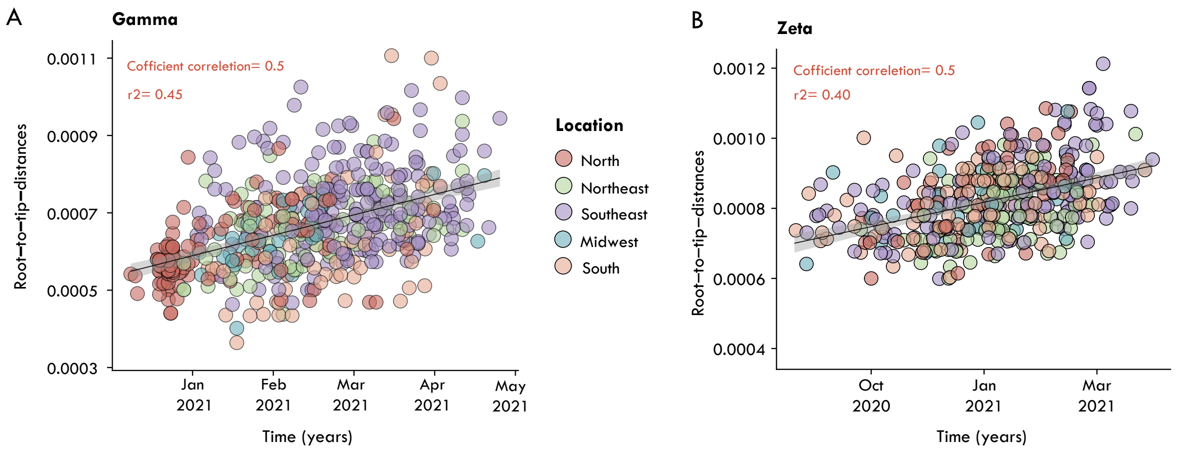

Supplement: Supplementary file 1 — Supplementary Figs. 1–5. [file 41564_2022_1191_MOESM1_ESM.pdf]
