# Supplementary material for: Genomic epidemiology of the SARS-CoV-2 epidemic in Brazil
Source: Nat Microbiol. 2022 Aug 18;7(9):1490–500. doi: 10.1038/s41564-022-01191-z (PMC9417986; doi:10.1038/s41564-022-01191-z)
Supplement: Supplementary file 2 — Reporting Summary [file 41564_2022_1191_MOESM2_ESM.pdf]

nature portfolio

Corresponding author(s): NMICROBIOL-21102473C

Last updated by author(s): Jul 16, 2022

Reporting Summary

Nature Portfolio wishes to improve the reproducibility of the work that we publish. This form provides structure for consistency and transparency in reporting. For further information on Nature Portfolio policies, see our Editorial Policies and the Editorial Policy Checklist.

Statistics

For all statistical analyses, confirm that the following items are present in the figure legend, table legend, main text, or Methods section.

n/a	Confirmed	
☐	☒	The exact sample size (*n*) for each experimental group/condition, given as a discrete number and unit of measurement
☐	☒	A statement on whether measurements were taken from distinct samples or whether the same sample was measured repeatedly
☒	☐	The statistical test(s) used AND whether they are one- or two-sided<br>*Only common tests should be described solely by name; describe more complex techniques in the Methods section.*
☒	☐	A description of all covariates tested
☒	☐	A description of any assumptions or corrections, such as tests of normality and adjustment for multiple comparisons
☐	☒	A full description of the statistical parameters including central tendency (e.g. means) or other basic estimates (e.g. regression coefficient) AND variation (e.g. standard deviation) or associated estimates of uncertainty (e.g. confidence intervals)
☒	☐	For null hypothesis testing, the test statistic (e.g. *F*, *t*, *r*) with confidence intervals, effect sizes, degrees of freedom and *P* value noted<br>*Give P values as exact values whenever suitable.*
☐	☒	For Bayesian analysis, information on the choice of priors and Markov chain Monte Carlo settings
☒	☐	For hierarchical and complex designs, identification of the appropriate level for tests and full reporting of outcomes
☒	☐	Estimates of effect sizes (e.g. Cohen's *d*, Pearson's *r*), indicating how they were calculated

Our web collection on statistics for biologists contains articles on many of the points above.

Software and code

Policy information about availability of computer code

Data collection	NA
Data analysis	R Studio, Seraphim, Beast, Tempest, Mafft, Aliview, Treetime, Pangolin, iqtree2, Genome Detective, Guppy

For manuscripts utilizing custom algorithms or software that are central to the research but not yet described in published literature, software must be made available to editors and reviewers. We strongly encourage code deposition in a community repository (e.g. GitHub). See the Nature Portfolio guidelines for submitting code & software for further information.

Data

Policy information about availability of data

All manuscripts must include a data availability statement. This statement should provide the following information, where applicable:

- Accession codes, unique identifiers, or web links for publicly available datasets
- A description of any restrictions on data availability
- For clinical datasets or third party data, please ensure that the statement adheres to our policy

All sequences that were generated and used in the present study are listed in table S1-S3 (accessible on the GitHub repository) along with their GISAID sequence IDs, dates of sampling, the originating and submitting laboratories and main authors. All input files (e.g. alignments or XML files), all resulting output files and scripts used in the study are shared publicly on GitHub (https://github.com/genomicsurveillance/Genomic_epidemiology_reveals_how_restriction_measures_shaped_the_SARS-CoV-2_epidemic_in_Brazil).

Human research participants

Policy information about studies involving human research participants and Sex and Gender in Research.

Reporting on sex and gender	Sequenced genomes were from samples collected from 2,023 females and 1,843 males (Table S1 and S2) with a median age of 41.72 years (range: 1 to 90 years of age).
Population characteristics	Sequenced genomes were from samples collected from 2,023 females and 1,843 males (Table S1 and S2) with a median age of 41.72 years (range: 1 to 90 years of age).
Recruitment	NA
Ethics oversight	This research was approved by the Ethics Review Committee of the Pan American Health Organization (PAHOERC.0344.01), the Federal University of Minas Gerais (CEP/CAAE: 32912820.6.1001.5149), the University of São Paulo (CEP/FZEA: 4.780.992) and the Blood Center of Ribeirão Preto (CEP/HCRP-FMRP: 50367721.7.1001.5440), and by the Paraguayan Ministry of Public Health and Social Welfare (MSPyBS/ S.G. no. 0944/18). The availability of these samples for research purposes during outbreaks of national concern is allowed under the terms of the 510/2016 Resolution of the National Ethical Committee for Research – Brazilian Ministry of Health (CONEP - Comissão Nacional de Ética em Pesquisa, Ministério da Saúde) that authorizes, without the necessity of an informed consent, the use of clinical samples collected in the Brazilian Central Public Health Laboratories to accelerate knowledge building and contribute to surveillance and outbreak response. The samples processed in this study were obtained anonymously from material exceeding that needed for routine diagnosis in Brazilian public health laboratories that belong to the public network within BrMoH.

Note that full information on the approval of the study protocol must also be provided in the manuscript.

Field-specific reporting

Please select the one below that is the best fit for your research. If you are not sure, read the appropriate sections before making your selection.

☒ Life sciences ☐ Behavioural & social sciences ☐ Ecological, evolutionary & environmental sciences

For a reference copy of the document with all sections, see nature.com/documents/nr-reporting-summary-flat.pdf

Life sciences study design

All studies must disclose on these points even when the disclosure is negative.

Sample size	A total of 3866 near-full genomes sequences from SARS-CoV-2 RT-qPCR positive samples were obtained as part of this study. SARS-CoV-2 sequencing spanned February 2020 to June 2021, sampled from 8 of the 27 Brazilian states (São Paulo=3309; Rio Grande do Sul=48; Paraná=55; Minas Gerais=80; Mato Grosso do Sul=36; Mato Grosso=51; Bahia=224) and one neighbouring country, Paraguay (n=63). Almost half of the sequences were from the Southeast of Brazil, consistent with it being responsible for most reported cases in the country (Fig. 1B) 6. The sequenced samples were collected from 2,023 females and 1,843 males (Table S1 and S2) with a median age of 41.72 years (range: 1 to 90 years of age). All tested samples contained sufficient viral genetic material (≥2ng/μL) for library preparation. For positive samples, PCR cycle threshold (Ct) values were on average 19.93 (range: 10.75 to 30). Sequences had a median genome coverage of 95% (range: 80 to 99.99) and average genome coverage was typically higher for samples with lower Ct values. Sequences were assigned to 39 different PANGO-lineages based on the proposed dynamic nomenclature for SARS-CoV-2 lineages and have been submitted to GISAID following the WHO guidelines (version 3.1.7, August 2021).
Data exclusions	To ensure the quality of the genome sequences generated in this study and to guarantee the highest possible phylogenetic accuracy, only genomes >29,000bp and <1% of ambiguities were considered.
Replication	NA
Randomization	NA
Blinding	NA

Reporting for specific materials, systems and methods

We require information from authors about some types of materials, experimental systems and methods used in many studies. Here, indicate whether each material, system or method listed is relevant to your study. If you are not sure if a list item applies to your research, read the appropriate section before selecting a response.

Materials & experimental systems

n/a	Involved in the study
☒	☐ Antibodies
☒	☐ Eukaryotic cell lines
☒	☐ Palaeontology and archaeology
☒	☐ Animals and other organisms
☒	☐ Clinical data
☒	☐ Dual use research of concern

Methods

n/a	Involved in the study
☒	☐ ChIP-seq
☒	☐ Flow cytometry
☒	☐ MRI-based neuroimaging
